# Associations of Voice Metrics with Postural Function in Parkinson’s Disease

**DOI:** 10.3390/life15010027

**Published:** 2024-12-30

**Authors:** Anna Carolyna Gianlorenço, Valton Costa, Walter Fabris-Moraes, Paulo Eduardo Portes Teixeira, Paola Gonzalez, Kevin Pacheco-Barrios, Ciro Ramos-Estebanez, Arianna Di Stadio, Mirret M. El-Hagrassy, Deniz Durok Camsari, Tim Wagner, Laura Dipietro, Felipe Fregni

**Affiliations:** 1 Neuromodulation Center and Center for Clinical Research Learning, Spaulding Rehabilitation Hospital, Massachusetts General Hospital, Harvard Medical School, 1575 Cambridge Street, Cambridge, MA 02115, USA; gianlorenco@ufscar.br (A.C.G.); valtoncosta@estudante.ufscar.br (V.C.); walterafmoraes@gmail.com (W.F.-M.); pgonzalezmego@mgb.org (P.G.); kevin.pacheco.barrios@gmail.com (K.P.-B.); 2 Laboratory of Neuroscience and Neurological Rehabilitation, Physical Therapy Department, Federal University of Sao Carlos, Rodovia Washington Luis, km 235, Sao Carlos 13565-905, SP, Brazil; 3 Faculty of Medicine, University of Sao Paulo, Avenida Doutor Arnaldo, 455, Sao Paulo 05508-220, SP, Brazil; 4 Highland Instruments, Inc., Cambridge, MA 02238, USA; paulo@highlandinstruments.com (P.E.P.T.); twagner@highlandinstruments.com (T.W.); lauradp@highlandinstruments.com (L.D.); 5 Unidad de Investigación para la Generación y Síntesis de Evidencias en Salud, Vicerrectorado de Investigación, Universidad San Ignacio de Loyola, Lima 15023, Peru; 6 Department of Neurology and Rehabilitation, University of Illinois at Chicago, 912 S Wood St., MC 796, Chicago, IL 60612, USA; cramoses@uic.edu; 7 Otolaryngology Unit, GF Ingrassia Department, University of Catania, 95121 Catania, Italy; ariannadistadio@hotmail.com; 8 Neurology Department, UMass Memorial, UMass Chan Medical School, Suite #301, 67 Belmont St., Worcester, MA 01605, USA; melhagrassy.neuromod@gmail.com; 9 Mayo Clinic, 200 1st St SW, Rochester, MN 55901, USA; drdenizdoruk@gmail.com; 10 Mindpath College Health, 948 Embarcadero del Norte #102, Isla Vista, CA 93117, USA; 11 Harvard/MIT Division of Health Sciences and Technology, Cambridge, MA 02139, USA

**Keywords:** Parkinson’s disease, voice disorder, acoustic analysis, smoothed cepstral peak prominence, posture, postural stability

## Abstract

Background: This study aimed to explore the potential associations between voice metrics of patients with PD and their motor symptoms. Methods: Motor and vocal data including the Unified Parkinson’s Disease Rating Scale part III (UPDRS-III), Harmonic–Noise Ratio (HNR), jitter, shimmer, and smoothed cepstral peak prominence (CPPS) were analyzed through exploratory correlations followed by univariate linear regression analyses. We employed these four voice metrics as independent variables and the total and sub-scores of the UPDRS-III as dependent variables. Results: Thirteen subjects were included, 76% males and 24% females, with a mean age of 62.9 ± 10.1 years, and a median Hoehn and Yahr stage of 2.3 ± 0.7. The regression analysis showed that CPPS is associated with posture (UPDRS-III posture scores: β = −0.196; F = 10.0; *p* = 0.01; R^2^ = 0.50) and UPDRS-III postural stability scores (β = −0.130; F = 5.57; *p* = 0.04; R^2^ = 0.35). Additionally, the associations between CPPS and rapid alternating movement (β = −0.297; *p* = 0.07), rigidity (β= −0.36; *p* = 0.11), and body bradykinesia (β = −0.16; *p* = 0.13) showed a trend towards significance. Conclusion: These findings highlight the potential role of CPPS as a predictor of postural impairments secondary to PD, emphasizing the need for further investigation.

## 1. Introduction

Parkinson’s Disease (PD) is the second most common neurodegenerative disease in the United States of America [1]. It is characterized by a range of motor and non-motor symptoms that progressively worsen over the course of the disease [2]. These symptoms can lead to disability, as well as a decreased quality of life, morbidity, and mortality [3,4,5,6]. Standard treatments currently include medication (primarily levodopa) and occupational/physical therapy. These treatments do not address disability caused by dysphonia, which affects 70% of PD patients and is described as one of their greatest deficits by 29% [7].

In addition to the cardinal motor symptoms of tremor, bradykinesia, rigidity, and postural instability, patients with PD are also affected by other motor symptoms that can impact physical and psychosocial function. Among these are hypokinetic dysphonia and dysarthria, prevalent dysfunctions that can manifest earlier than other motor symptoms in PD [8,9]. These are characterized by an impairment of the voice that may become perceptually “harsh and breathy” (dysphonia) and can be identified through acoustic analyses [10,11]. Research has revealed numerous anatomical and physiological abnormalities underlying speech and voice dysfunction in people with PD. These changes include laryngeal tremor, incomplete glottic closure, and the hypoadduction of vocal folds, which contribute to alterations in pitch, volume, and quality [11,12]. Additionally, the length of the vocal tract is altered due to changes in the control of the muscles in the larynx, tongue, and lips [13]. These motor changes result from the degeneration of the dopaminergic neurons in the basal ganglia, leading to the rigidity and bradykinesia of the vocal apparatus, which also contribute to appendicular and axial motor impairments [11]. However, it is believed that voice changes may occur earlier in the disease, as speech is a complex voluntary motor action and, therefore, may be more susceptible to slight degenerative alterations in the substantia nigra and the nigrostriatal circuits [14].

**Figure 1:**
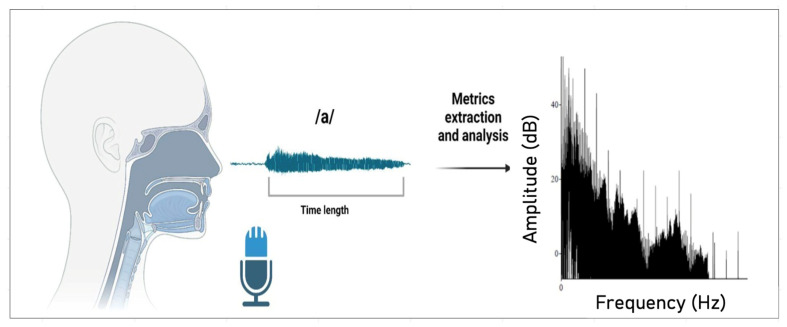
Illustration of the process for voice recording (sustained vowel /*a*/) and analysis of voice and speech parameters derived from the acoustic signal, from which several metrics can be extracted.

Dysphonia and other voice deviations can be identified by analyzing the main acoustic frequencies in the recorded signals during sustained phonation or continuous speech (see Figure 1). Speech and voice metrics are diverse, encompassing features of articulation, phonation, and prosody, and several of these metrics have been shown to be altered in PD [15]. However, the most used and validated voice-derived features are those obtained from analyzing the fundamental frequency (F0) of a speech signal (i.e., the average of vocal fold oscillations per second (Hz)) of the recorded signal, such as smoothed cepstral peak prominence (CPPS), as well as measures of perturbation or noise such as shimmer and jitter, Harmonic-to-Noise ratio, and other acoustic features [16,17,18,19,20]. This field has grown, and evidence now suggests that altered voice characteristics may hold more complex clues, potentially serving as predictors of physiological dysfunctions not directly related to dysphonia [21,22,23,24,25,26]. Therefore, voice-derived measurements hold potential for aiding in early diagnosis, serving as alternative measures of dysfunction, and monitoring therapeutic interventions.

Specifically, in the context of PD, some attempts have been made to assess the discriminative capabilities of voice-derived features in relation to motor phenotypes. Previous research has shown mixed evidence. For instance, the F0 is increased only in some features and with differences between men and women with PD [27]; increases in CPPS and prosody variables have been associated with worsening rest tremor and declines in axial function, particularly gait and postural stability [28,29,30,31,32,33]. Conversely, other studies have not found associations between voice and speech parameters—such as F0 range, pause duration, CPPS, disfluencies, and prosody—as well as tremor and other motor presentations [34,35]. However, the association of voice parameters with motor symptoms may be more specific to certain symptoms and sample characteristics. An overview of these findings is shown in Table 1, and description of the metrics are given in Table 2.

Therefore, it can be hypothesized that voice-related acoustic characteristics in PD may be associated with motor symptoms, especially posture-related motor aspects, as several of these symptoms might share concurrent dopaminergic and non-dopaminergic pathological mechanisms [11,37,38]. Thus, we aimed to explore potential associations between the voice metrics (the Harmonic–Noise Ratio (HNR)), jitter (local percentage), shimmer (local percentage), and CPPS of patients with PD and their motor symptoms.

## 2. Materials and Methods

### 2.1. Study Design

Our dataset was collected as part of the baseline assessments for a randomized controlled trial investigating non-invasive brain stimulation for the treatment of PD (ClinicalTrials.gov Identifier: NCT01615718) conducted at the Neuromodulation Center of the Spaulding Rehabilitation Hospital, Charlestown, MA, USA. Not all the patients examined herein entered the main trial; this dataset consists of those who underwent baseline voice assessments. This study was reviewed and approved by the Mass General Brigham Institutional Review Board. Written informed consent was obtained from all enrolled subjects before the start of the trial.

### 2.2. Participants

Inclusion criteria required subjects to have a diagnosis of PD from their clinician by either a letter or verification through their medical record; a research criteria of “possible” or “probable” PD, as defined by Gelb et al. [39]; be aged 40 or over; and have been on stable medications for at least 30 days. Exclusion criteria were any contraindication to non-invasive brain stimulation, features suggestive of other causes of parkinsonism/PD-plus syndromes, unstable medical conditions, and a history of DBS or ablation surgery. As above, thirteen subjects from the main study completed the voice assessments and were included in this analysis.

### 2.3. Study Variables

#### 2.3.1. Unified Parkinson’s Disease Rating Scale Part III (UPDRS-III)

The UPDRS-III is widely used in clinical and scientific settings, due to its comprehensive coverage of motor symptoms, and its strong clinimetric properties—reliability and validity [40]. This scale assesses fourteen motor aspects through clinician-administered evaluations, generating sub-scores for speech, facial expression, tremor at rest, postural tremor, rigidity, finger taps, hand movements, rapid alternating hand movements, leg agility, arising from chair, posture, gait, postural stability, and body bradykinesia [41], as well as an overall score (sum of all sub-scores).

#### 2.3.2. Vocal Recording and Measures

To extract voice characteristics, we recorded the subjects as they performed sustained vowel phonation [/a/] for at least 9 s. Voice samples were recorded and saved in WAV format, and only the medial 5 s of each recording were analyzed. The following four acoustic variables were extracted: the Harmonic–Noise Ratio (HNR), jitter (local percentage), shimmer (local percentage), and smooth cepstral peak prominence (CPPS). Voice analysis was performed with Praat version 6.4.12 (Institute of Phonetic Sciences, Amsterdam, Netherlands), according to the validated extraction method described by Sauder, Bretl, and Eadie (2017) [42].

#### 2.3.3. Voice Metrics

Four metrics were extracted from Praat processing (see Table 2). The Praat software version 6.4.12 applies a pre-emphasis filter at 50 Hz and uses Hann windowed high-pass and low-pass filters, which were set to a frequency range from 10 to 5000 Hz. CPPS was calculated as the difference in amplitude between the F0 peak and the baseline in the cepstral domain (inverse Fourier transformation). Both shimmer and jitter measure voice stability, which is the consistency and regularity of the acoustic waves over time, with higher values indicating increased voice instability. Shimmer represents the percentage of short-term amplitude variations across consecutive vocal cycles (sequence of opening and closing the top and bottom of the vocal folds) [43], while jitter represents the percentage of short-term variations in the fundamental frequency across consecutive vocal cycles [43]. The Harmonic-to-Noise Ratio (HNR), expressed in decibels (dB), assesses voice signal quality by quantifying the ratio of periodic sound energy to noisy (aperiodic) sound energy within the signal, with higher values indicating a clearer, more stable voice [18].

### 2.4. Statistical Analysis

Descriptive statistics were used to characterize the demographic and clinical variables, with mean and standard deviations (SDs) calculated for continuous variables when data were normally distributed, and medians and interquartile range (IQR) for not-normally distributed data. Frequencies (percentages) were used to describe categorical variables. Numerical variables’ distributions were assessed using histograms and the Shapiro–Wilk test. The statistical analyses included exploratory Pearson correlation tests for pairs of variables and further univariate linear regression analyses. In the latter, the main four voice metrics served as independent predictors (CPPS, shimmer, jitter, and HNR), with the total and sub-scores of the UPDRS-III as the dependent variables (including facial expression, speech, tremor at rest, postural tremor, rigidity, finger taps, hand movements, rapid alternating hand movements, leg agility, arising from chair, posture, gait, postural stability, and body bradykinesia). Higher UPDRS III sub-scores and total scores indicate worse function. To assess whether the results could be influenced by motor severity, the sample was divided into two groups based on the median total score of the UPDRS-III (below and above the median, indicating less severe and more severe motor symptoms). The four voice metrics were then compared between these groups using the Wilcoxon test. The significance level for all analyses was set at *p* < 0.05. All statistical analyses were performed using statistical software RStudio version 4.3.2 (Posit Software, PBC, Boston, MA, USA) with the “ggplot2” package version 3.5.1 for graph generation.

## 3. Results

### 3.1. Participant Characteristics

The sample included 13 subjects—10 males and 3 females—with a mean age of 62.9 ± 10.1 years and a median Hoehn and Yahr stage of 2.3 ± 0.7. The group consisted of two subjects classified at tremor-dominant, three as akinetic–rigid, and eight as mixed (classified according to Eggers et al. [44]). The mean UPDRS-III total score was 21.4 ± 11.0, with sub-scores shown in Table 3.

### 3.2. Exploratory Correlations

Statistically significant correlations were found between posture and CPPS (r = −0.71; 95% CI: −0.91, −0.23; F = 10.03; *p* = 0.01) and between postural stability and CPPS (r = −0.60; CI: −0.87, −0.04; F = 5.56; *p* = 0.04). No significant correlations were found between jitter, shimmer, HNR, and the overall (total) or sub-scores of the UPDRS-III, nor between CPPS and other sub-scores of the UPDRS-III.

### 3.3. Linear Regressions

The univariate linear regressions indicated that only CPPS was statistically significantly associated with two UPDRS-III sub-scores—posture and postural stability. The results of all other regression analyses are reported in Table 4.

### 3.4. UPDRS-III Posture and CPPS

**Figure 2:**
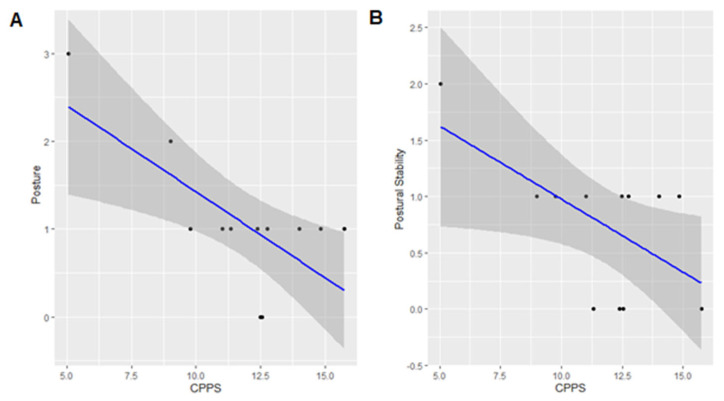
(**A**) Linear regression showing the association between CPPS (dB) and UPDRS-III posture sub-score. (**B**) Linear regression between CPPS (dB) and UPDRS-III postural stability sub-score.

The analysis showed that CPPS was associated with UPDRS-III posture scores (β = −0.196; SE = ±0.06; F = 10.0; *p* = 0.01; R^2^ = 0.50) (Figure 2A).

### 3.5. UPDRS-III Postural Stability and CPPS

The analysis showed that CPPS was associated with UPDRS-III postural stability scores (β = −0.130 ± 0.06; F = 5.57; *p* = 0.04; R^2^ = 0.35) (Figure 2B).

### 3.6. Comparison of Voice Measures by Motor Severity Levels

We stratified the patients according to the median UPDRS-III score and performed group comparisons using the Wilcoxon test. No statistically significant difference was found between groups for CPPS, jitter, shimmer, or HNR (W = 24, *p* = 0.39; W = 15, *p* = 0.61; W = 18, *p* = 0.25; W = 10, *p* = 0.76, respectively).

## 4. Discussion

We aimed to explore the associations between voice parameters and motor symptoms in PD. Our results indicated that CPPS was significantly associated with posture and postural stability. Lower CPPS values correlated with better posture and postural stability, independent of motor severity, as shown by the comparison between patients with less severe and more severe motor symptoms. This finding suggests that poor vocal stability correlates with poor postural stability and posture, and vice versa.

CPPS is a validated objective measure used to identify vocal instabilities, assess voice quality, and quantitatively characterize dysphonia severity [16,18,45]. Several studies have utilized this acoustic metric to investigate various conditions. In PD, CPPS has been used to measure dysphonia, with some studies also exploring its clinical relevance to cognitive status and motor impairments [18,28,29,30,33,46,47]. However, the relationship between this voice measure and motor symptoms remains incompletely understood. One hypothesis posits that PD may begin in the gut and olfactory bulb, potentially affecting early cognitive function due to lesions in the dorsal motor nucleus of the vagus nerve, with pathology spreading to the brainstem and eventually reaching the forebrain as it progresses. Thus, PD progression is linked to the spread of α-synuclein pathology through specific neural pathways, rather than being confined to the substantia nigra [48]. Additionally, studies have shown that decreased CPPS is associated with worse rest tremor, while increases in other voice variables, like prosody and phonation, have been linked to impaired gait and postural instability [28,29,30]. Our findings align with these previous studies and highlight the potential role of CPPS as a quantitative measure and potential predictor of postural impairment in PD.

These findings support the idea of a direct functional relationship between posture and speech. During speech, various muscles involved in the control of posture and respiration are activated, altering the proprioceptive and coordination, and directly impacting voice features, like volume and pitch [38,49,50,51]. Research indicates that this relationship is bilateral as altered body posture is also associated with dysphonia, suggesting that one could potentially predict the other [37,38]. In our study, we observed a relationship between CPPS and posture and postural stability, which might reflect a common pathogenesis pathway in the disease progression. Pathologic changes in the striatal circuits lead to alterations in the motor control of both vocal folds and axial/appendicular muscles. These could be independent consequences of dopaminergic loss, or the result of an interaction, where vocal changes lead to postural alterations or vice versa [9,11]. Given that voice alterations like dysphonia are reported to be a very early manifested symptom in PD [8,9], CPPS warrants greater attention in PD clinical research. Could CPPS serve as a biomarker for postural instability? Future studies should explore this possibility, as it could aid in preventing functional physical decline at diagnosis and provide an objective measure, especially when the direct assessment of postural instability is not feasible.

Interestingly, we did not find significant correlations between CPPS and other motor aspects assessed using UPDRS-III, including speech. This may be due to the limited sensitivity of the speech sub-item, as it is subjectively evaluated and considers multiple aspects of speech simultaneously, such as diction, volume, modulation, clarity, and speed, from the assessor’s perspective. The sample size and the homogeneity of the disease stage in our sample may also have limited our analysis sensitivity. Additionally, we did not observe significant correlations between other voice metrics (i.e., shimmer, jitter, and HNR) and motor symptoms, underscoring the potential role of CPPS as a sensitive marker for postural impairment. The diversity of metrics and analytic methods may also contribute to overall divergent findings.

The field would significantly benefit from the standardization of computerized methods and the validation of specific metrics in PD, with CPPS emerging as a promising candidate [17]. Voice recordings, in particular, offer a feasible and effective approach for monitoring symptom progression and treatment responses. This method is especially relevant in both telerehabilitation and clinical settings, where mobile recording devices and home-based applications can facilitate continuous, real-time assessments [52,53]. By leveraging such technologies, clinicians could gain valuable insights into individual patient trajectories, leading to more personalized and timely interventions. Further studies are needed that include control groups consisting of healthy individuals and/or patients with advanced-stage PD.

### 4.1. Impact of the Study Results on the Field of Otolaryngology

Voice and speech disorders are generally managed by otolaryngologists and speech and language pathologists. Voice changes may be related to abnormal physiology, such as spasmodic dysphonia or tremor, organic causes like glottic cancer, or neurological diseases [54]. Although studies have identified an increase in F0, this alteration alone cannot serve as a “pure” indicator that the neurological disorder of hearing loss—which also affects F0—is prevalent in this population [55]. Assessing CPPS may help to better determine the underlying cause of voice changes and aid in identifying patients with PD.

It is important to emphasize that the study found a correlation between CPPS and stability, and that good stability and normal voice production require proper muscle functionality. Both the larynx and glottic plan contain intrinsic and extrinsic muscles, so any alteration in signal transmission within central motor control, including that associated with PD, can manifest as voice changes. Otolaryngologists and speech and language pathologists should be aware that voice changes, particularly low CPPS, might be an early biomarker of PD.

### 4.2. Limitations of the Study

The main limitation of our study is the sample size, which restricts the generalization of our findings and the sensitivity of our analyses. For instance, our analyses included patients with only mild to moderate motor impairment, some of whom did not exhibit dysphonia or other related symptoms. Despite this, the study presents valuable findings that could be further explored in future, well-powered studies. Such studies should control for relevant population characteristics, including disease duration, stage, age, and gender, as well as other relevant clinical variables such as body composition, tabagism, alcohol consumption, physical activity level, respiratory function, and fatigue.

## 5. Conclusions

These preliminary findings highlight the associations between a voice metric (CPPS) and posture and postural stability in patients with PD. Controlled studies with larger sample sizes and multivariate analyses are needed to confirm these results, investigate their clinical implications, and explore the potential utility of these associations while accounting for possible confounders. Additionally, this work raises important considerations regarding the relationship between posture and voice alterations, including etiological and pathological interactions that are important for understanding symptom manifestations and monitoring their progression in PD. Thus, this study contributes to the field by underscoring the potential use of CPPS as a potential metric for assessing motor symptoms, such as postural function, beyond its role in voice assessment in PD.

## Figures and Tables

**Table 1:** Summary of previous evidence on the association between voice and speech metrics and motor symptoms in PD.

Authors/ Year	Sample	Goal	Motor Symptoms	Main Voice Metrics	Analyses	Results
Burk & Watts, 2018 [28]	32 PD (ON period, H&Y: 2.62-2.75), 10 HC	Differentiate tremor and non-tremor phenotypes.	Tremor (UPDRS and self-reporting)	CPP (dB)	Sustained vowel (/a/) and connected speech (Computerized Speech Lab - Pentax Medical, Montvale, NJ)	Tremor dominant exhibited lower CPP than non-tremor subjects and control.
Goberman, 2005 [29]	9 PD (ON period, H&Y)	Examine the associations between voice and motor variables.	Factored UPDRS-III: Axial function/gait, rest tremor, rigidity, bradykinesia, postural tremor	F_0_ and F_0_ SD	Sustained vowel (/i/, /u/, /a/, /ae/) and continuous speech (Computerized Speech Lab software - Kay Elemetrics).	F_0_ SD associated with axial and non-axial motor symptoms.
Skodda et al., 2011 [30]	169 PD (ON period, H&Y: 2.51), 64 HC	Explore correlations of prosodic and motor symptoms.	Total UPDRS-III and sub-scores	F_0,_ SD, and variation range	Continuous speech (Praat, Version 5.1 - Institute of Phonetic Sciences, University of Amsterdam)	Mean F_0_ associated to axial UPDRS-II sub- scores; F_0_ variability reduced in PD.
Dias et al., 2016 [32]	50 PD (ON period, H&Y: 2.71-3.18)	Correlate speech impairment and motor symptoms.	UPDRS-III: tremor, rigidity, bradykinesia, axial impairment	Formant frequency values F1 and F2	Sustained vowel (/a/, /i/, /u/), continuous and spontaneous speech (Praat software v5.3.30 - Phonetic Sciences, University of Amsterdam)	Associations between the metrics and axial, rigidity, and bradykinesia sub- scores.
Gillivan-Murphy, Miller & Carding, 2019 [33]	30 PD (OFF period), 28 HC	Examine correlations of voice tremor and disease variables.	UPDRS-III total score	Voice tremor rate (rate, periodicity, variation, and amplitude of F_0_)	Sustained vowel (/a/) (Multi-Dimensional Voice Program, Computerized Speech Laboratory)	Only the rate of amplitude voice tremor correlated negatively with UPDRS-III; voice disability did not correlate with voice tremor; rate of tremor higher in PD than HC.
Brown & Spencer, 2020 [34]	27 PD (ON period)	Investigate whether acoustic dysarthria aligns with non-tremor and tremor-dominant profiles.	MDS-UPDRS-III (classification of tremor profiles)	F_0_ range (Hz), average pause duration, CPPS (dB)	Continuous speech (Praat - Boersma & Weenink, 2017 and Adobe Audition Version 9.0)	No differences were observed between the motor profiles.
Skodda et al., 2009 [35]	50 PD (ON period), 50 HC	Analyze changes in speech over time (up to 79 months) and correlated with motor impairment.	UPDRS-III total score	F_0_, SD, and variation range (Hz)	Continuous speech (Praat - Phonetic Sciences, University of Amsterdam)	No association between the changes in the vocal metrics and changes in UPDRS-III.

PD, Parkinson’s disease; HC, healthy control; H&Y, Hoehn and Yahr scale (mean); CPP, cepstral peak prominence; UPDRS, Unified Parkinson’s Disease Rating Scale (III: part III); F_0_, fundamental frequency; SD, standard deviation; MDS-UPDRS-III, Movement Disorder Society-sponsored UPDRS update; CPPS, smoothed cepstral peak prominence.

**Table 2:** Definitions and descriptions of voice metrics.

	Units	Objective	Clinical Relevance	Interpretation
**CPPS**	decibels (dB)	To measure the regularity and periodicity of the voice signal, focusing on the fundamental frequency and its prominence in the cepstrum.	Useful for detecting subtle changes in voice periodicity and diagnosing voice disorders affecting vocal fold vibrations.	High CPP suggests a highly regular voice signal and good vocal quality, while low CPP suggests aperiodicity, which may be associated with voice disorders.
**Harmonic–Noise Ratio (HNR)**	decibels (dB)	To measure the relative amount of periodic (harmonic) energy to aperiodic (noise).	Provides a broader measure of overall voice quality and is useful for diagnosing voice disorders that introduce noise.	High HNR indicates a clear and stable voice, while low HNR suggests potential pathologies.
**Shimmer**	milliseconds (ms) or percentages (%)	To measure variations in the amplitude.	Important parameter for assessing vocal quality and health.	Low shimmer translates to loudness stability, while high shimmer suggests an unhealthy voice.
**Jitter**	decibels (dB) or percentages (%)	To measure variations in the fundamental frequency.	Used to diagnose and monitor voice disorders.	Low jitter reflects pitch stability, while high jitter indicates possible disorders.
**Fundamental Frequency (F0)**	hertz (Hz)	To measure the rate at which vocal folds vibrate, representing the pitch of the voice.	Vital for understanding pitch control, voice quality, and diagnosing voice disorders related to pitch regulation.	A low F0 indicates a slower vibration of the vocal folds, producing a lower-pitched voice.

This table provides descriptions of the voice metrics and their relevance in clinical practice [18,32,36].

**Table 3:** Descriptive data for clinical and voice measures.

UPDRS-III variables	Mean ±SD
Speech	1.6 ± 0.9
Facial expression	1.2 ± 1.0
Rigidity	3.2 ± 2.0
Finger tapping	1.8 ± 1.2
Hand movements	1.9 ± 1.5
Alternating movements	1.8 ± 1.5
Leg agility	1.8 ± 1.7
Posture	1.1 ± 0.8
Gait	0.8 ± 0.7
Postural stability	0.7 ± 0.6
Bradykinesia	1.5 ± 1.0
UPDRS-III	18.0 (13.0)
Arising from chair	0 (0)
Kinetic Tremor	2.0 (1.0)
Tremor	1.0 (3.0)
**Voice metrics**	Mean (±SD)
Jitter	0.67 ± 0.37
Shimmer	6.56 ± 2.12
CPPS	11.75 ± 2.86
HNR	21.11 ± 5.94

UPDRS-III, Unified Parkinson Disease Rating Scale, part III; CPPS, smoothed cepstral peak prominence; HNR, harmonic-to-noise ratio.

**Table 4:** Univariate linear regression analyses of voice metrics and UPDRS-III sub-scores and overall score (dependent variables).

UPDRS-III	Voice	β-Coefficient	95% CI		SE	*p* Value
Postural stability	CPPS	−0.130	−0.252	−0.007	0.055	0.040 *
	Jitter	0.326	−0.719	1.371	0.453	0.493
	Shimmer	−0.041	−0.223	0.141	0.079	0.618
	HNR	0.015	−0.050	0.080	0.028	0.612
Posture	CPPS	−0.196	−0.334	−0.058	0.061	0.010 *
	Jitter	0.293	−0.952	1.538	0.539	0.602
	Shimmer	−0.013	−0.230	0.205	0.094	0.896
	HNR	0.015	−0.063	0.092	0.033	0.675
Speech	CPPS	−0.108	−0.307	0.091	0.089	0.255
	Jitter	0.247	−1.795	2.289	0.885	0.787
	Shimmer	−0.001	−0.354	0.352	0.153	0.995
	HNR	−0.007	−0.134	0.119	0.054	0.894
Gait	CPPS	−0.088	−0.240	0.065	0.068	0.228
	Jitter	−0.088	−1.353	1.178	0.548	0.877
	Shimmer	0.066	−0.146	0.277	0.091	0.494
	HNR	−0.054	−0.118	0.010	0.027	0.088
Tremor	CPPS	0.274	−0.431	0.978	0.316	0.407
	Jitter	−1.131	−8.303	6.041	3.110	0.726
	Shimmer	0.378	−0.826	1.583	0.522	0.489
	HNR	−0.265	−0.654	0.123	0.168	0.154
Body bradykinesia	CPPS	−0.161	−0.379	0.056	0.097	0.129
	Jitter	0.858	−1.195	2.912	0.890	0.363
	Shimmer	0.046	−0.325	0.417	0.160	0.783
	HNR	−0.034	−0.165	0.096	0.056	0.560
Facial expression	CPPS	−0.086	−0.321	0.149	0.105	0.433
	Jitter	0.190	−2.170	2.550	1.023	0.857
	Shimmer	−0.160	−0.545	0.225	0.167	0.367
	HNR	0.041	−0.101	0.182	0.061	0.525
Rapid alternating	CPPS	−0.297	−0.631	0.038	0.150	0.076
	Jitter	−0.860	−4.311	2.592	1.496	0.582
	Shimmer	−0.105	−0.705	0.495	0.260	0.697
	HNR	0.016	−0.200	0.232	0.093	0.866
Kinetic tremor	CPPS	0.076	−0.242	0.395	0.143	0.604
	Jitter	−0.037	−3.273	3.198	1.403	0.980
	Shimmer	−0.042	−0.598	0.513	0.240	0.864
	HNR	0.048	−0.147	0.243	0.084	0.586
Rigidity	CPPS	−0.356	−0.815	0.102	0.205	0.114
	Jitter	−1.573	−5.857	2.711	1.858	0.422
	Shimmer	0.259	−0.480	0.998	0.320	0.443
	HNR	−0.177	−0.411	0.057	0.101	0.119
Finger tapping	CPPS	−0.085	−0.391	0.221	0.137	0.548
	Jitter	−1.337	−4.067	1.393	1.183	0.292
	Shimmer	−0.250	−0.713	0.212	0.200	0.247
	HNR	0.013	−0.167	0.194	0.078	0.871
Hand movements	CPPS	−0.184	−0.547	0.178	0.162	0.283
	Jitter	−1.899	−5.036	1.239	1.361	0.200
	Shimmer	−0.310	−0.856	0.236	0.236	0.227
	HNR	0.046	−0.165	0.258	0.091	0.627
Leg agility	CPPS	−0.144	−0.558	0.269	0.185	0.455
	Jitter	−1.200	−4.986	2.585	1.642	0.486
	Shimmer	−0.232	−0.877	0.413	0.279	0.431
	HNR	−0.051	−0.287	0.186	0.102	0.635
Arising from chair	CPPS	-	-	-		-
UPDRS-III total	CPPS	−1.560	−4.113	0.993	1.146	0.203
	Jitter	−6.210	−30.212	17.792	10.409	0.567
	Shimmer	−0.405	−4.611	3.800	1.823	0.830
	HNR	−0.395	−1.869	1.079	0.639	0.554

CPPS, smoothed cepstral peak prominence; HNR, harmonic-to-noise ratio (HNR); SE, standard error. Values of the sub-score “arising from chair” were not included in the regression analysis because all patients scored 0 (normal or no problems). * *p* < 0.05.

## Data Availability

No data associated with our study have currently been deposited in a publicly available repository.
